# Quantitative Pleural Fluid Echogenicity for Differentiating Transudative From Exudative Pleural Effusions

**DOI:** 10.7759/cureus.97242

**Published:** 2025-11-19

**Authors:** Alaa A El-Dakkak, Mona El-Hoshy, Maged Hassan

**Affiliations:** 1 Pulmonology Department, Faculty of Medicine, Alexandria University, Alexandria, EGY

**Keywords:** echogenicity, exudates, pleural effusion, thoracic ultrasound, transudates

## Abstract

Introduction

Thoracic ultrasonography (TUS) is more sensitive than chest radiography for identifying pleural fluid. It aids in differentiating between transudates and exudates by observing fluid echogenicity and septations.

This study aimed to assess whether quantitative estimation of pleural effusion echogenicity, using a novel index termed pleural fluid relative echogenicity (PFRE), can non-invasively differentiate exudates from transudates.

Methods

The study recruited 140 patients with pleural effusion. TUS stills of pleural effusion were stored as grayscale images for correlation with biochemical results. Two distinct methods were employed to quantify echogenicity. The first involved selecting two regions of interest (ROI), one within the liver and another within the effusion. The second method involved measuring the quantitative echogenicity of the overall area of pleural effusion and comparing it with that of the liver in the same image.

PFRE was calculated as the ratio of pleural fluid echogenicity to liver echogenicity. The predictive ability of PFRE in distinguishing transudates from exudates was evaluated, taking Light’s criteria as the gold standard.

Results

A total of 140 patients were included. Per Light’s criteria, 44 patients had transudative effusion and 96 had exudative effusion. Using the ROI method, the median PFRE was 0.26 (0.15-0.44) in transudates and 0.55 (0.35-0.72) in exudates (p<0.001). Positive correlation was found between the effusion protein levels and PFRE (r=0.303, P<0.001). There was a negative correlation between glucose levels and PFRE (r= 0.176, P= 0.039).

The area under the curve (AUC) for PFRE to differentiate transudates from exudates was 0.77 (95% confidence interval (CI) 0.69 to 0.84). A cut-off of ≤0.236 for PFRE showed a sensitivity of 47.73%, a specificity of 90.62%, a positive predictive value of 70%, and a negative predictive value of 79.1% to predict transudative effusion. The overall area method showed less accurate results.

Conclusion

PFRE can differentiate between transudative and exudative effusion non-invasively with a moderate degree of accuracy.

## Introduction

Thoracic ultrasonography (TUS) has greatly transformed the management of pleural disease. Besides being more sensitive than chest radiography in identifying pleural effusion, it allows the characterization of pleural fluid and pleural space and facilitates safe pleural intervention [1,2]. Thoracic ultrasound is more accurate than chest computed tomography (CT) in identifying septations in a pleural effusion [3,4].

Determining whether the pleural effusion is an exudate or a transudate is the first stage in the diagnostic workup. Light's criteria are considered the gold standard for such assessments [5,6]. Thoracic ultrasonography helps in distinguishing transudates from exudates, as transudates are often anechoic because they typically lack any substance that might reflect ultrasound waves [7]. Exudates, on the other hand, may exhibit a heterogeneously echogenic pattern with spinning debris, septations, fronds, or strands [8-10]. Exudates that contain a large number of cells, such as hemothorax or empyema, may have a uniform echogenicity [11-13]. However, studies have shown that these qualitative greyscale ultrasound features, when taken separately, are not sensitive enough to reliably differentiate between exudative and transudative effusions [11,14]. A recent study that employed a composite score of qualitative variables, including echogenicity, reported much improved accuracy [15].

Quantitative echogenicity is an emerging imaging biomarker used in the diagnosis of multiple diseases in different organs. Quantitative echoic indices (EIs) have been shown to help in the detection of malignant thyroid nodules as well as the assessment of renal cortical structural changes [16,17]. There are only a few studies investigating the diagnostic utility of quantitative sonographic echogenicity in the management of pleural effusions [18,19].

Three studies are reported in the literature that specifically examined the utility of quantitative echogenicity in the characterization of pleural effusion. In the first study, the authors used image processing software to assess pleural fluid echogenicity based on pixel density. The cohort comprised 83 patients whose TUS images were obtained retrospectively, and the authors combined quantitative analysis with qualitative criteria. The results indicated significantly higher pleural fluid echogenicity, as determined by pixel density, in exudative effusions [18]. This study included effusions with septations, which is a marker of exudative effusion. Furthermore, the use of pixel density as an absolute measure would not account for variations in sonographic window settings. Therefore, the true impact of the study results was difficult to ascertain.

The second study introduced the hypoechogenicity index (HI), calculated as the ratio of the mean echo level of the rib pixels to that of the pleural effusion pixels in a cohort of 61 patients using the same image processing software used in the study by Soni et al. [18]. The results demonstrated a correlation between the HI and various biochemical parameters, including lactate dehydrogenase (LDH), cell count, pH, and the mean number of effusion pixels [19]. It is noteworthy that this study did not aim to differentiate between exudative and transudative effusions and included septated effusion in the echogenicity measurements, which impacted the ability to ascertain the exact value of quantitative echogenicity.

This current study aimed to prospectively assess the utility of quantitative pleural effusion echogenicity for distinguishing between exudative and transudative effusions in nonseptated or minimally septated effusions, avoiding previous research limitations. A novel imaging biomarker termed pleural fluid relative echogenicity (PFRE), comparing pleural fluid echogenicity to liver echogenicity of each patient independently, was used to control variations in sonographic window settings among patients.

## Materials and methods

This is a diagnostic accuracy study using prospectively collected data. The study protocol was approved by the Ethics Committee of Alexandria Faculty of Medicine (ref: 000I2098). Written informed consent was obtained from all participants.

The study recruited patients referred to the Chest Diseases Department at Alexandria University Hospitals for diagnostic or therapeutic thoracentesis. Patients with septated effusions on TUS, those who underwent a recent ipsilateral pleural procedure (within 7 days), and those with established fatty liver disease on ultrasound or CT were excluded.

Ultrasound still pictures of the pleural effusion were saved as grayscale images. For uniformity of TUS parameters (image depth, gain, and focus position), in patients with right-sided effusion, one image was saved for the effusion and the liver. For left-sided effusion, two images were saved using the same depth and gain: one for the effusion and another for the liver. All patients were scanned by the same operator using the convex probe (frequency range 1.3-5.7 MHz) of a DP-10 Mindray ultrasound (US) machine (Shenzhen Mindray Bio-medical Electronics Co., China).

**Figure 1:**
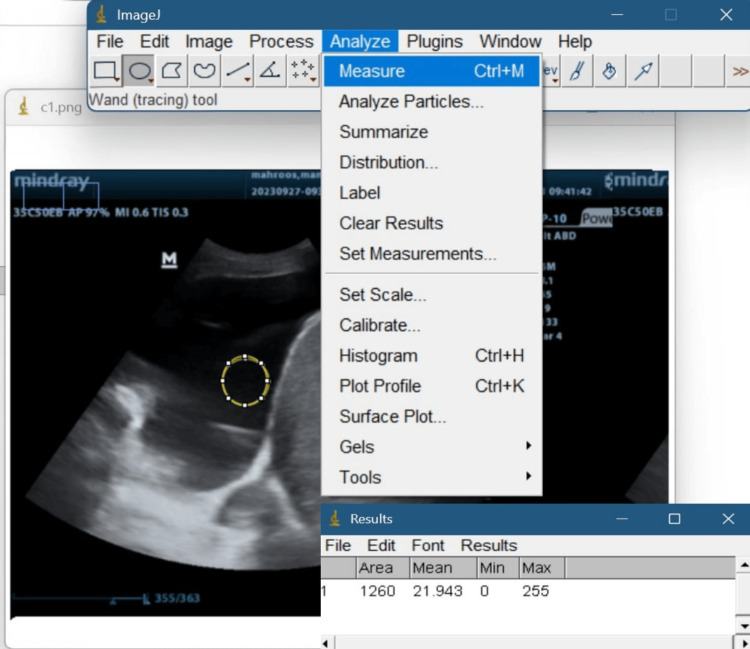
Image analysis was conducted using ImageJ software

TUS images were transferred to a computer to be analyzed by an open-access image processing software (ImageJ; National Institutes of Health). ImageJ allows analysis of grayscale images by imparting values ranging from 0 (black) to 255 (white) according to their pixel density (Figure 1). The mean pixel density of an area of interest in the image was used as a measurement of its quantitative echogenicity [19].

**Figure 2:**
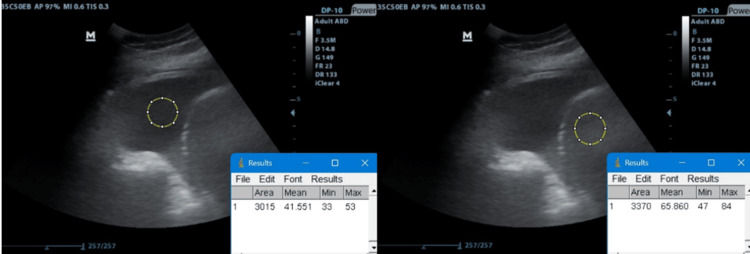
Detecting pleural fluid relative echogenicity for right-sided effusion by selecting the ROI for the pleural fluid and for the liver in the same photo ROI: regions of interest

In patients with right-sided effusion, two regions of interest (ROI) were chosen in the same photo: one within the liver and another within the effusion (to compare liver vs. effusion echogenicity using the same TUS settings) (Figure 2). In cases with left-sided effusion, two images were saved, one for the effusion and another for the liver, and one ROI was chosen in each photo.

**Figure 3:**
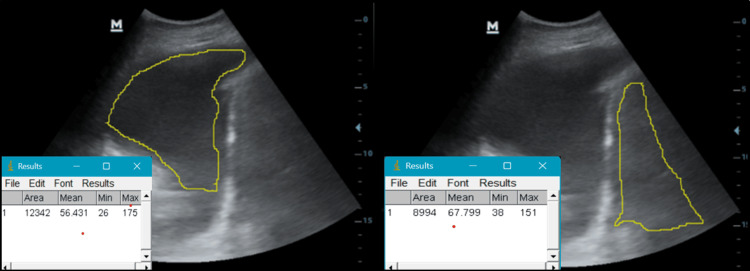
Detecting pleural fluid relative echogenicity using the overall area method

A second method was applied to estimate quantitative echogenicity by marking the overall area of the pleural effusion in the image and comparing it to the overall area of the liver in the image (as opposed to choosing an ROI) (Figure 3).

Pleural fluid relative echogenicity (PFRE) was calculated as the ratio of the pleural fluid echogenicity (measured as the mean pixel density) to that of the liver. This ratio was used to overcome the sonographic window difference (i.e., chest wall impedance) between patients. The PFRE of transudates and exudates was calculated.

The gold standard criteria for defining exudates and transudates were Light's criteria (pleural fluid protein: serum protein ratio > 0.5, or pleural fluid: serum LDH ratio > 0.6, or pleural fluid LDH > two-thirds upper limits of the laboratory's normal serum LDH) [20]. In addition, correlations between PFRE and different biochemical and cellular components of the pleural fluid were explored.

Pleural effusion etiology was determined based on clinical factors in addition to biochemical, microbiological, and cytological analysis of pleural fluid.

Statistical analysis

Data were analyzed using SPSS software package version 20.0 (Armonk, NY: IBM Corp) and MedCalc version 17 (MedCalc Software Ltd, Ostend, Belgium). Categorical data were presented as numbers and percentages. The chi-square test was used for comparing categorical variables. Alternatively, the Monte Carlo correction test was applied when more than 20% of the cells had an expected count of less than 5. Quantitative data were expressed as mean + standard deviation or median (interquartile range) according to data normality. The Mann-Whitney or Student's t-test was used to compare qualitative data between the two groups. Receiver operating characteristic curve (ROC curve) analysis was used to examine the diagnostic ability of PFRE to differentiate between transudates and exudates. Spearman correlation was used to examine correlations between PFRE and pleural fluid biochemical components. Differences were deemed significant when the P value was < 0.05.

## Results

The study recruited 140 patients from September 2022 to January 2024. Characteristics of the patients and pleural effusions are summarized in Table 1. A total of 68 patients (48.6%) were males. The mean age was 53.8±15.7 years. Ninety-six patients (68.5%) had an exudative pleural effusion, while 44 patients (31.54%) had transudates. Regarding the etiology of the pleural effusion, malignancy was the predominant cause, accounting for 43.6% of cases, followed by infections at 15.7%. Cardiac, hepatic, and renal failure were the underlying etiologies in 12.1%, 12.1%, and 7.1% of the included patients, respectively.

**Table 1:** Demographic data and pleural fluid characteristics of recruited patients SD: standard deviation, Lt: left, Rt: right, TB: tuberculosis, LDH: lactate dehydrogenase, N: number, IQR: interquartile range, mg/dL: milligram per deciliter, g/dL: gram per deciliter, U/L: unit per liter

Demographic Data	Transudate (N=44)	Exudative (N=96)
Age (Years)	60.1±10.4	50.9±16.9
Mean ± SD.		
Sex	23 (52.3%)	45 (46.9%)
Males		
Females	21 (47.7%)	51 (53.1%)
Effusion side	12 (27.3%)	9 (9.4%)
Bilateral		
Lt	6 (13.6%)	38 (39.6%)
Rt	26 (59.1%)	49 (51.0%)
Effusion etiology	0	2 (2.1%)
Autoimmune disease		
Heart failure	17 (38.6%)	0
Hepatic failure	17 (38.6%)	0
Chylothorax	0	1 (1%)
Pleural infection	0	22 (22.9%)
Malignancy	4 (9.1%)	57 (59.4%)
Nonspecific pleuritic	0	1 (1%)
Pseudomeig syndrome	0	1 (1%)
Renal failure	6 (13.6%)	4 (4.2%)
TB	0	8 (8.3%)
Clinical investigations	199.2±139.1	113.7±90.4
Pleural fluid glucose (mg/dL)		
Mean ± SD.		
Pleural fluid protein (g/dL)	2.1±.7	4.7±1
Mean ± SD.		
Pleural fluid LDH (U/L)	82 (69-129.3)	397 (218.5-925.2)
Median (IQR)		

**Figure 4:**
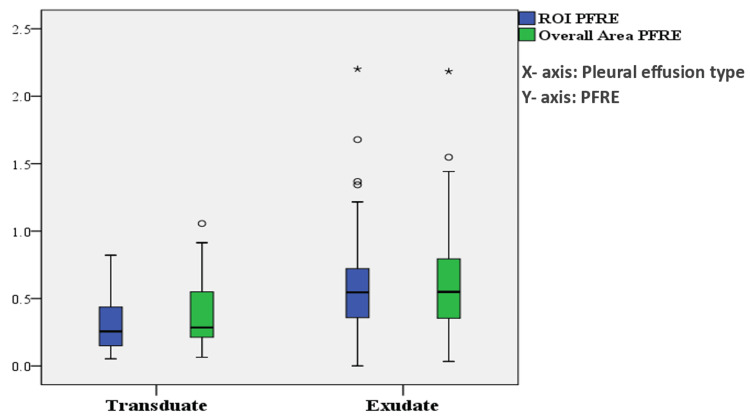
Comparing PFRE using the ROI and overall area methods in exudative and transudative effusions ROI: region of interest, PFRE: pleural fluid relative echogenicity

Regarding pleural fluid echogenicity using the ROI method, the median PFRE for the whole studied sample was 0.44 (0.27-0.64). The median PFRE was 0.26 (0.15-0.44) for transudates and 0.55 (0.35-0.72) for exudates (P<0.001). Using the overall area method, the median PFRE for the whole sample was 0.47 (0.28-0.72). The median PFRE was 0.29 (0.21-0.55) for transudates and 0.55 (0.36-0.79) for exudates (P<0.001) (Table 2, Figure 4).

**Table 2:** Comparing PFRE using ROI and overall area methods in exudative and transudative effusions ROI: region of interest, PFRE: pleural fluid relative echogenicity, IQR: interquartile range, N: number, U: Mann-Whitney test

Method	Transudate (N=44)	Exudative (N=96)	Test of sig
ROI	0.26 (0.15-0.44)	0.55 (0.35-0.72)	(U=953, P<0.001)
Median (IQR)			
Overall area	0.29 (0.21-0.55)	0.55 (0.36-0.79)	(U=-1214, P<0.001)
Median (IQR)			

**Figure 5:**
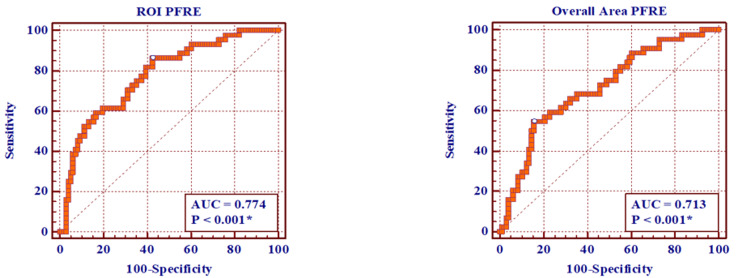
ROC curves for PFRE using the ROI and overall area methods to differentiate between exudative and transudative effusion ROI: region of interest, PFRE: pleural fluid relative echogenicity, AUC: area under the curve

Regarding the diagnostic ability of PFRE to differentiate between exudates and transudates, the ROI method had an area under the curve (AUC) of 0.77 (95% confidence interval (CI) 0.69 to 0.84, p<0.001), while the overall area method had an AUC of 0.71 (95% CI 0.63 to 0.79, p<0.001). Regarding the ROI method, a cutoff point of ≤0.2363 for diagnosing transudative pleural effusion showed a sensitivity of 47.7%, a specificity of 90.6%, a positive predictive value of 70%, and a negative predictive value of 79.1%. The overall area method showed less accurate results with a cutoff point of ≤0.2927, showing a sensitivity of 54.6%, a specificity of 84.4% with a positive predictive value of 61.5%, and a negative predictive value of 80.2% (Table 3, Figure 5).

**Table 3:** Diagnostic ability of PFRE using the ROI and overall area methods to differentiate between exudative and transudative effusion (N = 140) ROI: region of interest, PFRE: pleural fluid relative echogenicity, AUC: area under the curve, CI: confidence interval, PPV: positive predictive value, NPV: negative predictive value

NPV (95% CI)	PPV (95% CI)	Specificity (95% CI)	Sensitivity (95% CI)	cutoff point	P-value	AUC (95% CI)	Method
79.1 (70.3 - 86.3)	70.0 (50.6 - 85.3)	90.6 (82.9 - 95.6)	47.7 (32.5 - 63.3)	≤0.2363	<0.001	0.77 (0.69 to 0.84)	ROI PFRE
80.2 (71.1 - 87.5)	61.5 (44.6 - 76.6)	84.4 (75.5 - 91.0)	54.6 (38.8 - 69.6)	≤0.2927	<0.001	0.71 (0.63 to 0.79)	Overall Area PFRE

There was a positive correlation between the pleural fluid protein levels and ROI PFRE (r=0.303, P <0.001) and a negative correlation between pleural fluid glucose levels and ROI PFRE (r=-0.176, P=0.039) (Table 4).

**Table 4:** Correlations between PFRE using the ROI and overall area methods with lab investigations of the studied sample ROI: region of interest, PFRE: pleural fluid relative echogenicity, LDH: lactate dehydrogenase

Method	Test of sig (P)	ROI PFRE	Overall area PFRE	Glucose	LDH	Protein
ROI PFRE	r	1	0.898	-0.176	0.113	0.303
	P		<0.001	0.039	0.183	<0.001
Overall area PFRE	r	0.898	1	-0.153	0.115	0.242
	P	<0.001		0.072	0.176	0.004

## Discussion

This study aimed to evaluate the feasibility of using a quantitative measurement of pleural effusion echogenicity to non-invasively differentiate between exudative and transudative pleural effusions. We employed a novel metric, the PFRE, comparing the sonographic echogenicity of a pleural effusion to that of the liver by using a computer-based software (ImageJ) to analyze images exported from the ultrasound device. The findings revealed that PFRE had a moderate degree of accuracy in determining the pleural effusion nature, whereby the PFRE showed high specificity but low sensitivity in distinguishing transudative from exudative effusions.

In the study by Soni et al., the pixel density (which was used to measure quantitative echogenicity of pleural fluid) also had high specificity and low sensitivity for determining the nature of the pleural effusion [18]. This pattern suggests that measuring echogenicity as an absolute value for characterizing pleural fluid will inherently underdiagnose exudative pleural effusion. This is due to the well-documented phenomenon that some transudative effusions exhibit echogenicity [14].

We found that the PFRE positively correlated with pleural fluid protein levels. This is conceptually understandable, as the most likely explanation for these echogenic debris on TUS is that they represent proteinaceous material. This finding is not similar to that of Kalkanis et al., who utilized the hypoechogenicity index to assess pleural effusion echogenicity [19]. In their study, pleural fluid echogenicity showed a significant correlation with pleural fluid LDH levels, but not protein. This discrepancy between Kalkanis et al. and the present study can be attributed to including septations during pleural fluid echogenicity measurement in the former study. Septations, which are highly echogenic structures, tend to be seen in inflammatory effusions with high LDH levels.

We employed two methods (the ROI method and the overall area method) to estimate PFRE. While both methods yielded comparable results, the ROI method tended to consistently show better specificity results. This may be attributed to the more common occurrence of artifacts at the TUS image peripheries included in the overall area method in comparison to an ROI chosen at the center of the effusion.

Some studies have attempted to employ composite scores using qualitative US parameters plus other imaging findings to non-invasively identify the nature of a pleural effusion with very good results [15,18,19]. Among the TUS features included in these scores were fluid septations and their complexity, which strongly point to a non-transudative nature of the effusion. Our included cohort was predominantly made up of patients who had nonseptated effusions to understand specifically the value of pleural fluid echogenicity (in isolation) in pointing to the nature of the pleural fluid. This would be particularly useful in situations where other sonographic features, such as septations or nodularity, are lacking. We think that the aforementioned scores would not perform very well in patients such as those included in the present study.

This study has some limitations that are important to consider when interpreting results. This was a single-center study with a relatively small sample size. A single model of US machines was used; therefore, other machines with different specifications may show different results. Finally, there was no validation cohort to confirm the study results.

PFRE demonstrates promise as a non-invasive biomarker for both risk stratification and disease severity assessment. Also, it highlights the prognostic potential of PFRE across a spectrum of pleural effusion etiologies. Future research should aim to incorporate diverse methodologies and parameters, such as imaging techniques and clinical parameters, to provide a more solid and accurate framework for effusion characterization. This can overcome the limitations observed in our study and improve the efficacy of non-invasive methods for diagnosing and managing pleural effusions in clinical practice.

## Conclusions

In conclusion, pleural fluid relative echogenicity can help determine the nature of pleural effusion (exudative vs. transudative) in non-septated effusions with a moderate degree of accuracy. Future studies are needed to validate the findings of this study and to examine other potential uses of quantitative echogenicity in areas such as prognostication and predicting treatment response in different pleural diseases.
